# Immunomodulatory effect of polysaccharides isolated from *Lonicera japonica* Thunb. in cyclophosphamide-treated BALB/c mice

**DOI:** 10.1016/j.heliyon.2022.e11876

**Published:** 2022-11-25

**Authors:** Tao Zhang, Hongping Liu, Pengcheng Ma, Jian Huang, Xinyu Bai, Ping Liu, Lei Zhu, Xun Min

**Affiliations:** a Department of Laboratory Medicine, Affiliated Hospital of Zunyi Medical University, Zunyi 563003, China; b School of Laboratory Medicine, Zunyi Medical University, Zunyi 563006, China; c School of Pharmacy, Zunyi Medical University, Zunyi, Guizhou, 563006, China

**Keywords:** *Lonicera japonica* Thunb., Polysaccharide, Uronic acid, Immunomodulatory, Cyclophosphamide, Functional food

## Abstract

*Lonicera japonica* (*L. japonica*) is utilized as foods and healthy drink around the world. Until now, the immunomodulatory activity of polysaccharides from *L. japonica* (LJP) is little studied. In our previous work, LJP was fractionated to a neutral component (LJP-N) and an acidic component (LJP-A) by a DEAE-Cellulose column, and LJP-N was a starch-like polysaccharide and LJP-A was a pectic polysaccharide. In this study, LJP-N and LJP-A were investigated for their immunomodulatory effect, and the protective effect of these two polysaccharide fractions against immune injury by cyclophosphamide (CTX) in BALB/c mice was evaluated. Results showed that when compared with CTX group, LJP fractions, especially for LJP-A (200 mg kg^−1^) can enhance the immune function, improve atrophy of the lymphoid organs thymus and spleen (the increased approximately 1.8- and 1.6-fold), increase the phagocytic function of macrophages (increased approximately 1.7-fold), increase the secretion of cytokines (the levels of IL-6, IL-2, and TNF-α increased approximately 2.5-, 2.0-, and 1.4-fold) and immunoglobulins levels (the levels of IgM and IgG increased approximately 1.2- and 1.7-fold) in serum, and enhance the cytotoxic activity of NK cells (increased approximately 3.5-fold). Taken together, the present results suggest that LJP-A, rich in uronic acid, with a molecular weight of 400 kDa, may be a potential candidate as the functional foods.

## Graphical abstract

**Figure:**
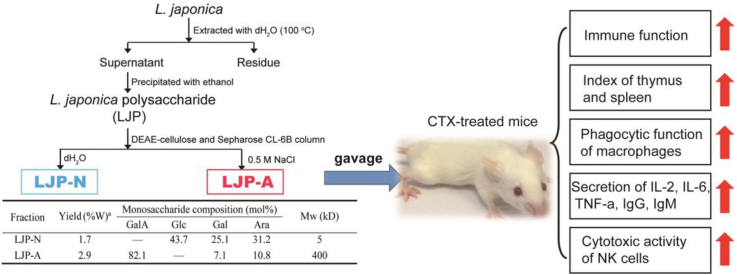

## 1 Introduction

Polysaccharides are complex macromolecules, widely distributed in nature. Owing to its positive actions on the human body and the number of biomedical effects, polysaccharide has been widely applied in the food fields for a long time (Korolenko et al., 2020; Minzanova et al., 2018; Nešić et al., 2020). Some polysaccharides have been discovered as potential chemical entities showing good anticancer effects, which can be exploited as alternatives to cancer chemotherapeutic agents used in clinical fields with minimal toxic side effects (Gopu and Selvam, 2020; Khan et al., 2019). Some dietary polysaccharides are considered prebiotic substances, which can significantly ameliorate symptom in type 2 diabetes mellitus, including hyperglycemia, hyperlipidemia, *etc* (Ganesan and Xu, 2019; Xiong et al., 2022; Zhang et al., 2019).

*Lonicera japonica* Thunb. (*L. japonica*) is named as ‘‘Jinyinhua” in China, which possess wide pharmacological activities, such as antiviral, antioxidant, antipancreatic cancer, *etc*. *L. japonica*, commonly used as healthy beverage and food additives around the world, can provide many nutrients for human body. Polysaccharide, the major active components of *L. japonica,* generally have a rather low toxicity, and it is usually readily soluble in water (Bai et al., 2020a, Bai et al., 2020b; Lin et al., 2016; Liu et al., 2019a, Liu et al., 2019b; Wang et al., 2014; Yang et al., 2019). Until now, the immunomodulation activity of polysaccharides from *L. japonica* (LJP) is little studied. In our previous work, we studied the effect of LJP on ovalbumin-induced allergic rhinitis (AR) mice. After LJP treatment, AR symptoms were relieved, and the levels of IL-17, IL-1β, TNF-α, and IgE in serum were significantly decreased. LJP was a mixture, which contained several types of polysaccharide domains, and the major active component in LJP on immunomodulation effect was unknown yet. So LJP was purified to a neutral component LJP-N and an acidic component LJP-A. LJP-N was a starch-like glucan, while LJP-A was a pectic polysaccharide (Bai et al., 2020a, Bai et al., 2020b; Zhang et al., 2020). In this study, LJP-N and LJP-A were investigated for its immunomodulatory effect *in vivo*, and the protective effect of these two polysaccharide fractions against immune injury by cyclophosphamide (CTX) in BALB/c mice was evaluated.

## 2 Materials and methods

### 2.1 Materials

*L. japonica* (voucher No. LJT-2018-001) was obtained from Suiyang county local market, Zunyi city, Guizhou province. CTX was purchased from Shanghai shifengbiol Co. (China). RPMI 1640 and fetal bovine serum (FBS) were purchased from Gibco (USA). 3-(4,5-dimethylthiazol-2-yl)-2,5-diphenyltetrazolim bromide (MTT) were purchased from Sigma (USA). IL-6 ELISA kit, IL-2 ELISA kit, TNF-a ELISA kit, IgG total ELISA kit, and IgM ELISA kit of mouse were all purchased from Beijing 4A Biotech Co., Ltd (China). Cell culture products were purchased from Thermo Fisher Scientific Co., Ltd (USA). Analytical reagent of others were all purchased in China.

### 2.2 Animal

Sixty female BALB/c mice (20–22 g, 6–8 week) were purchased from Changsha Tianqin Biotechnology Co., Ltd (China; certificate no. SCXK (Xiang) 2019-0014). Housing and breeding of mice were performed in strict compliance with Animal Care and Use Guidelines in China, and the experimental procedures were approved by the animal Ethics Committee of Zunyi Medical University (2017-2-098).

### 2.3 Preparation of LJP-N and LJP-A

**Figure 1:**
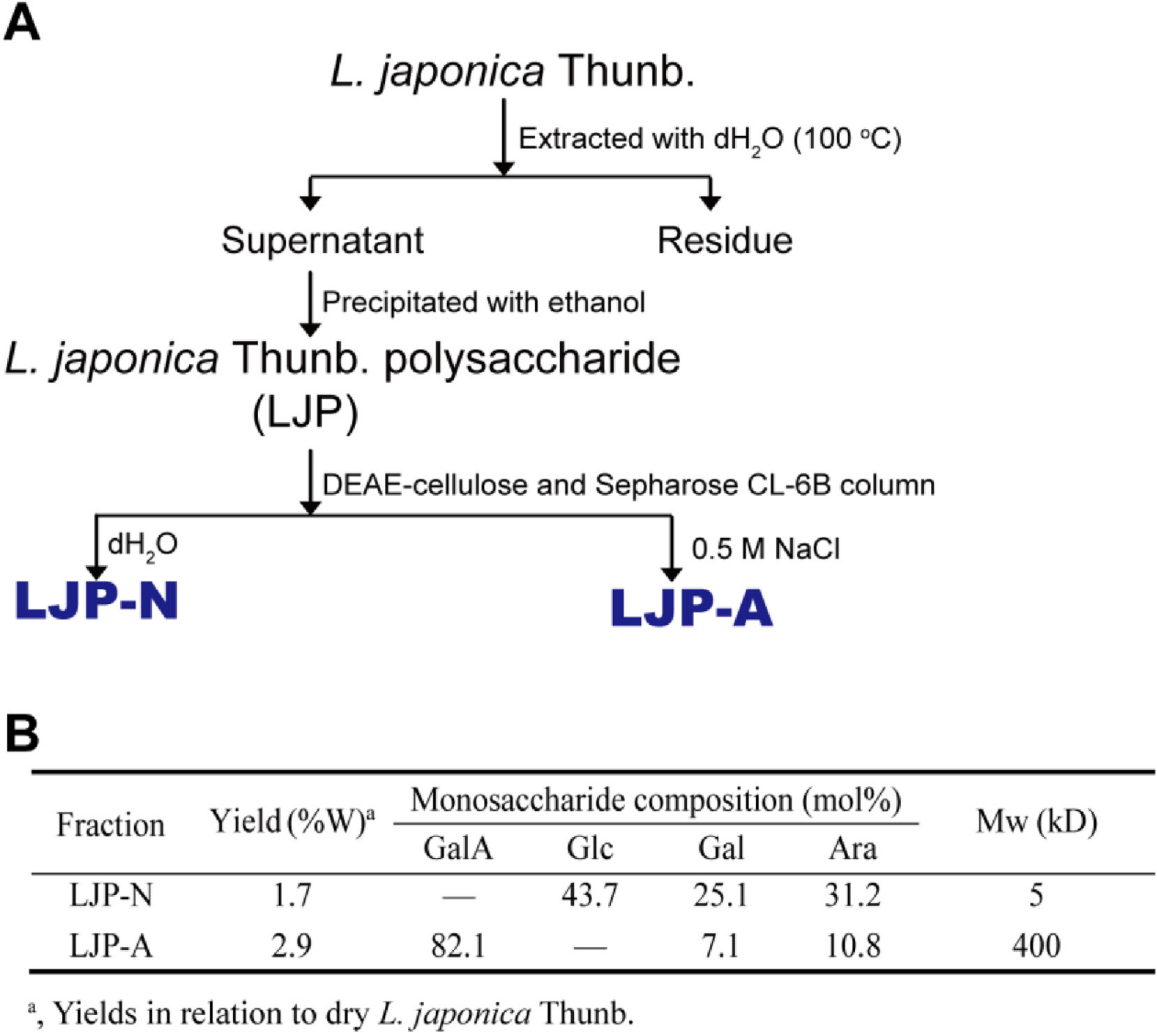
Preparation and primary structures of LJP-N and LJP-A. (A) Extraction and fractionation scheme; (B) monosaccharide composition and molecular weight.

LJP-N and LJP-A were purified from *L. japonica* as described in our previous studies (Liu et al., 2019a, Liu et al., 2019b; Zhang et al., 2020). In brief, *L. japonica* (500 g) was extracted with dH_2_O at 100 °C, and crude polysaccharide (LJP) was collected by add ethanol to the aqueous up to 80%. LJP was isolated sequentially by DEAE-Cellulose and Sepharose CL-6B chromatographies as showed in Figures 1A and B. LJP was applied to a DEAE-Cellulose column, which was eluted with dH_2_O and 0.5 M NaCl, respectively. And the elutes obtained were isolated using Sepharose CL-6B molecular sieve chromatography to give LJP-N and LJP-A two components. LJP-N was a starch-like glucan as well as some arabinogalactan and/or arabinan domains, with a molecular weight of 5.4 kDa; LJP-A was a pectic polysaccharide, mainly contained galacturonan with some galactan and/or arabinan domains, with a molecular weight of 400 kDa, approximately.

### 2.4 CTX-induced mice model and treatment

Mice were divided into 6 groups (10 mice per group). Normal mice group was defined as negative control (NC). Days 1–4, the rest mice were injected with CTX (70 mg kg^−1^ d^−1^) by intraperitoneally manner to prepare the immunosuppressive mice model by the published method (Wang et al., 2011, 2018). Day 5 to day 14, mice were administered intragastrically: NC group, 0.9% NaCl; CTX group, 0.9% NaCl; four polysaccharide groups, 50 mg kg^−1^ d^−1^ or 200 mg kg^−1^ d^−1^ LJP-N or LJP-A dissolved in 0.9% saline. 24 h after last treatment, the mice were weighted, blood was sampled and sacrificed via cervical dislocation. Thymus and spleen were both removed and weighted immediately, and organ indexes were calculated as the organ weight divided by body weight.

### 2.5 Determination of cytokines and immunoglobulins in serum

Twenty-four hours after the last treatment, serum was collected by enucleating eyeball. The concentrations of TNF-α, IL-6, and IL-2 as well as IgM and IgG were detected by ELISA kits based on the instruction of manufacturer.

### 2.6 Determination of phagocytic index

A carbon clearance assay was applied to measure the activity of macrophage cells, which was performed on three mice (per group) according to the published method (Wang et al., 2011). Tail vein of mice were all injected with 100 μL 10^−1^ g^−1^ of diluted India ink. Blood specimens were collected at the time of 2 min (t_1_) and 10 min (t_2_) from retinal venous plexuses, 20 μL of which was mixed with 2 mL of 0.1% Na_2_CO_3_. OD_600_ (A_1_, A_2_) was measured with 0.1% Na_2_CO_3_ as blank control, and A_1_, A_2_ was for t_1_, and t_2_, respectively. Meanwhile, body weight (B), spleen weight (S) and liver weight (L) of mice were all recorded, and the calculation equation of phagocytic index was as given in Eqs. (1) and (2):

(1) Y = (lgA_1_ − lgA_2_) (t_2_ − t_1_)^−1^

(2) Phagocytic index = Y ^1/3^ × B/(L + S)

### 2.7 Determination of NK cell cytotoxic activity

YAC-1 cells (mouse lymphoma cells) were cultured 24 h and adjusted to 4 × 10^4^ cells mL^−1^. Spleens were treared into small pieces in a germ-free condition, and a sterile sieve mesh was applied to filter the suspension to collect cell suspension. The cells were treated with red blood cells lysis buffer followed by washing with cold RPMI-1640 twice, and then the cells were adjusted to 2 × 10^5^ cells/mL in RPMI-1640 (10% FBS). A 100 μL of spleen cells (2 × 10^5^ cells/mL) and YAC-1 cells (4 × 10^4^ cells/mL) were incubated into 96-well plates using RPMI1640 medium containing 10% FBS. After 5-hour incubation in a 5% CO2 incubator at 37 °C, 5 mg/mL MTT (20 μL) were added to each well for additional 4 h incubation and subjected to MTT assay. NK cell activity was calculated as given in Eq. (3):

(3) NK activity (%) = (A_T_ − (A_S_ − A_E_)) A_T_^−1^ × 100%

where A_T_ is absorbance value at 490 nm of target cells control, A_S_ is absorbance value of test samples, and A_E_ is absorbance value of effector cells control.

### 2.8 Statistical analysis

All data were showed as mean ± S.D. Multiple t-tests were conducted to determine the statistical significance among means by using R language (R Development Core Team, 2014; www.R-project.org). A value of *P* < 0.05 was considered statistically significant.

## 3 Results and discussion

### 3.1 Effect of LJP fractions on body weight and immune organ indices in immunosuppressed mice

**Figure 2:**
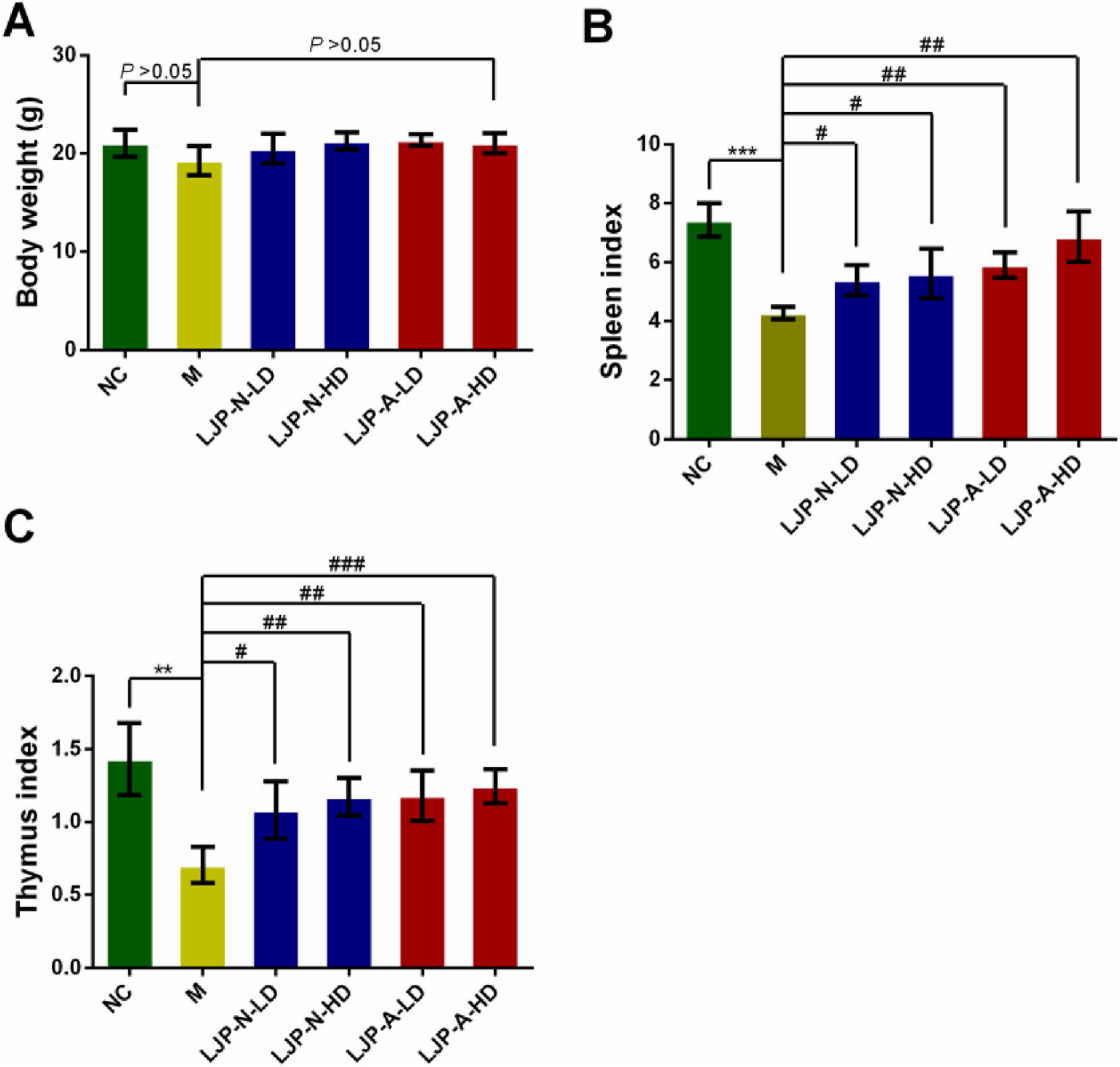
Effects of LJP fractions on the body weight and the immune organ indices of immunosuppression mice. (A) body weights of CTX group, NC group, and drug treatment group showed on Day 14; (B) spleen index; (C) thymus index. ∗∗, *P* < 0.01 and ∗∗∗, *P* < 0.001 *vs*. NC; #, *P* < 0.05, ##, *P* < 0.01 and ###, *P* < 0.001 *vs.* M. LD, 50 mg kg^−1^, HD, 200 mg kg^−1^.

Body weights of mice were decreased after CTX injection continuously compared with NC group mice (*P* > 0.05). After 10-day treatment, LJP-N and LJP-A could both improve body weights gradually, but there was no significance (*P* > 0.05) (Figure 2A). As shown in Figure 2B and C, spleen index and thymus index of mice treated with CTX at dose of 70 mg kg^−1^ body weight decreased significantly when compared with NC group mice, suggesting that the immmunosuppressed modeling was built successfully. Compared with CTX model mice, spleen indices of mice treated with LJP-N or LJP-A of 50 mg kg^−1^ or 200 mg kg^−1^ increased significantly with a dose-dependent manner. Besides, LJP-A showed more significant at the low concentration (*P* < 0.01) than LJP-N at the high concentration (*P* < 0.05). Compared with the CTX model mice, thymus indices of mice treated with LJP-N or LJP-A of 50 mg kg^−1^ or 200 mg kg^−1^ also increased significantly.

### 3.2 Effects of LJP fractions on cytokines in immunosuppressed mice

**Figure 3:**
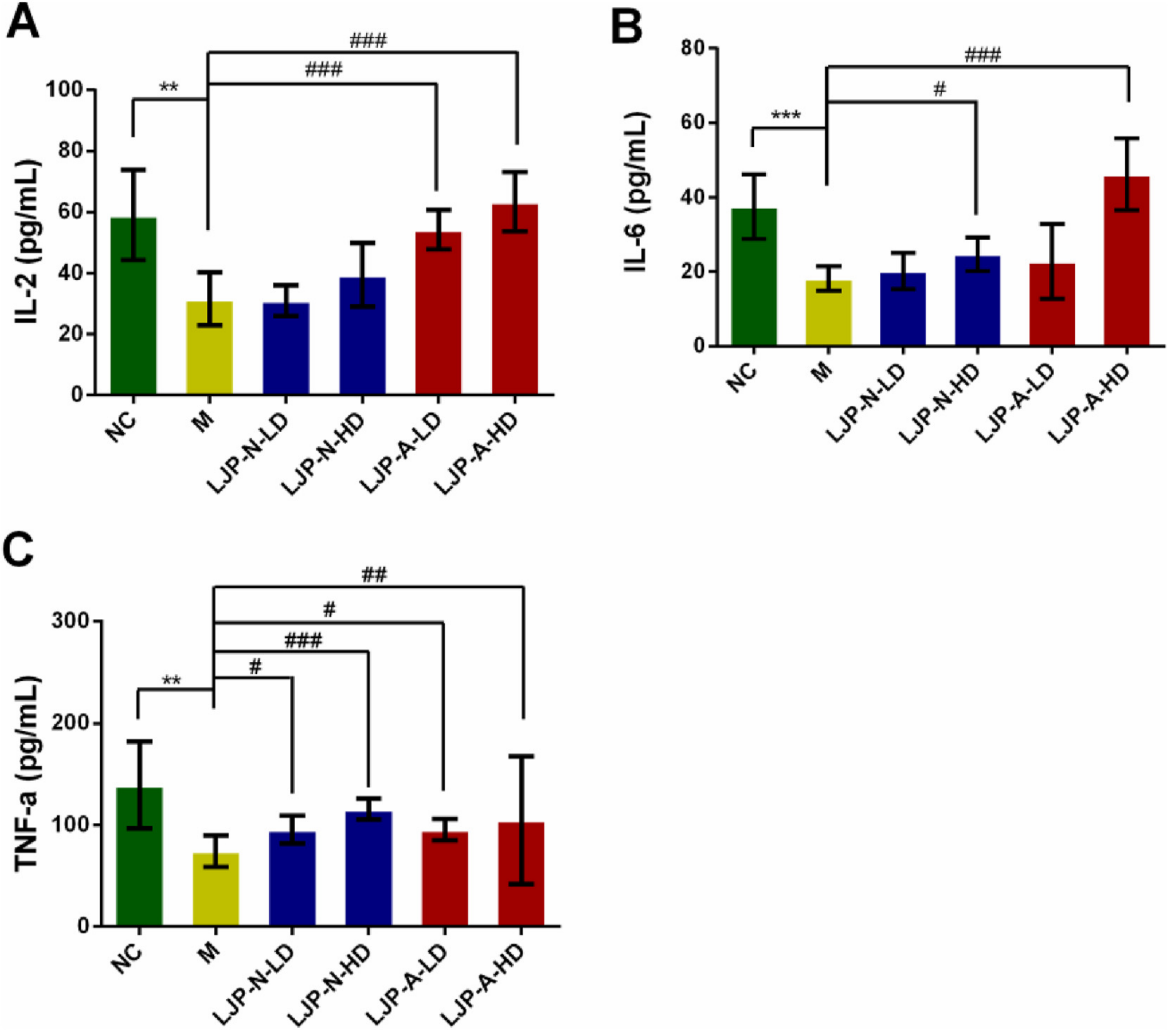
Mice were treated with LJP-N or LJP-A (50 mg kg^−1^ and 200 mg kg^−1^) for 10 days. The levels of IL-2 (A), IL-6 (B) and TNF-α (C) in serum of CTX-induced immunosuppression mice were measured via ELISA assay. ∗∗, *P* < 0.01 and ∗∗∗, *P* < 0.001 *vs*. NC; #, *P* < 0.05, ##, *P* < 0.01 and ###, *P* < 0.001 *vs.* M. LD, 50 mg kg^−1^, HD, 200 mg kg^−1^.

Cytokine plays essential roles in the human immune system to regulate the nature of the response, which is secreted by CD4^+^ helper T lymphocytes (Liu et al., 2016, 2019a,b). To further elucidate the immunomodulating effect of LJP-N and LJP-A, the serum level of TNF-α, IL-6, and IL-2 from CTX-treated mice were measured by ELISA assay. Compared with NC group mice, the levels of TNF-α, IL-6, and IL-2 were all reduced in immunosuppressed mice, significantly. After LJP-N or LJP-A administration, there were both a dose-dependent increase of IL-2 concentration (Figure 3A). Besides, compared with CTX model group, the concentration of IL-2 (Figure 3A), IL-6 (Figure 3B) and TNF-α (Figure 3C) from LJP-A treated group (200 mg kg^−1^) detected more cytokines and the concentrations reached 63.47, 46.23 and 104.86 pg/mL, which were 2.0-, 2.5- and 1.4-fold increases, respectively.

Different cytokines commonly work together and affect the synthesis of other cytokines. Th1 cells and Th2 cells secrete IL-2, TNF-α and IL-6 could promote cell-mediated immune responses and humoral or allergic responses (Chen et al., 2010; Fan et al., 2013; Hu et al., 2016). IL-2 plays an important role in immunomodulation and mediates cellular immunity, which can drive T-cell growth, augment NK cytolytic activity, induce the differentiation of regulatory T cells, and mediate activation-induced cell death (Huang et al., 2013; Letsch and Scheibenbogen, 2003; Liao et al., 2011; Zhao and Ashraf, 2015). IL-6, one of the most important immune and inflammatory mediators among the pro-inflammatory cytokines, can regulate diverse cell functions (such as proliferation and differentiation of B and T cells) (Sobota et al., 2008). TNF-α, also a key molecule to keep human health, can induce the expression of other immunoregulatory mediators (Qiao et al., 2016). These results indicate that LJP fractions, LJP-A especially, may have regulatory effects on the enhancement of immunity through the cytokine secretions.

### 3.3 Effects of LJP fractions on immunoglobulins in immunosuppressed mice

**Figure 4:**
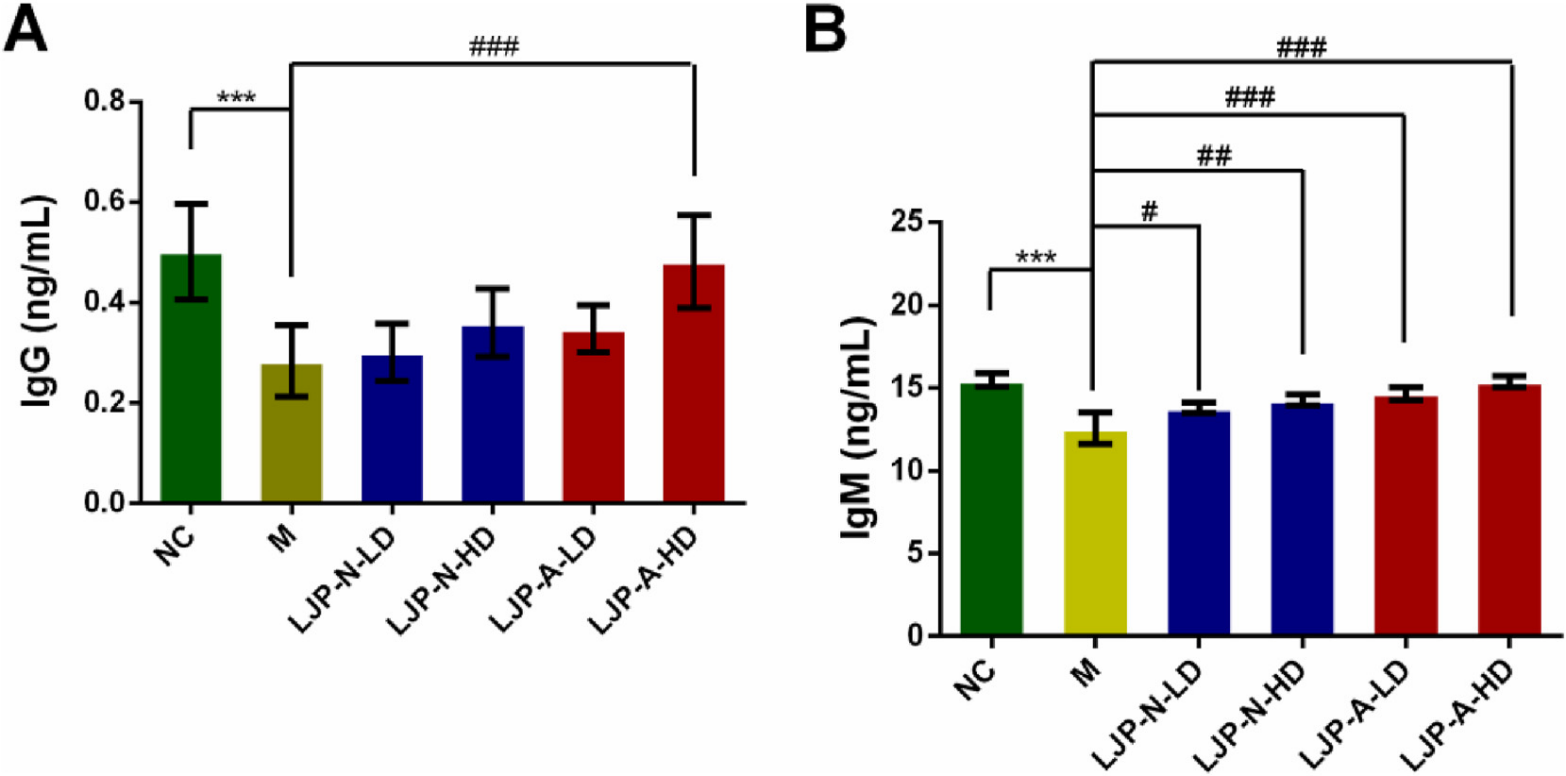
Mice were treated with LJP-N or LJP-A (50 mg kg^−1^ and 200 mg kg^−1^) for 10 days. The levels of IgG (A) and IgM (B) in serum of CTX-induced immunosuppression mice were measured via ELISA assay. ∗∗∗, *P* < 0.001 *vs*. NC; #, *P* < 0.05, ##, *P* < 0.01 and ###, *P* < 0.001 *vs.* M. LD, 50 mg kg^−1^, HD, 200 mg kg^−1^.

To elucidate the effects of LJP fractions, the levels of IgG and IgM in serum from immunosuppressed mice were measured by the marketed ELISA kits. The results showed that CTX could inhibit these Ig levels, while LJP-A could significantly increase them at the dose of 200 mg kg^−1^. Especially at concentration of 200 mg kg^−1^, the levels of IgG (Figure 4A) and IgM (Figure 4B) were increased approximately 1.7- and 1.2-fold compared with model group, respectively (*P* < 0.001). Whereas cotreatment with LJP-N showed a trend of increased IgG, but it was not significant. Thus, humoral immunity could be involved in LJP-A-mediated immunomodulatory effects in CTX-induced immunosuppressed mice.

### 3.4 Effects of LJP fractions on macrophage phagocytosis in CTX-treated mice

**Figure 5:**
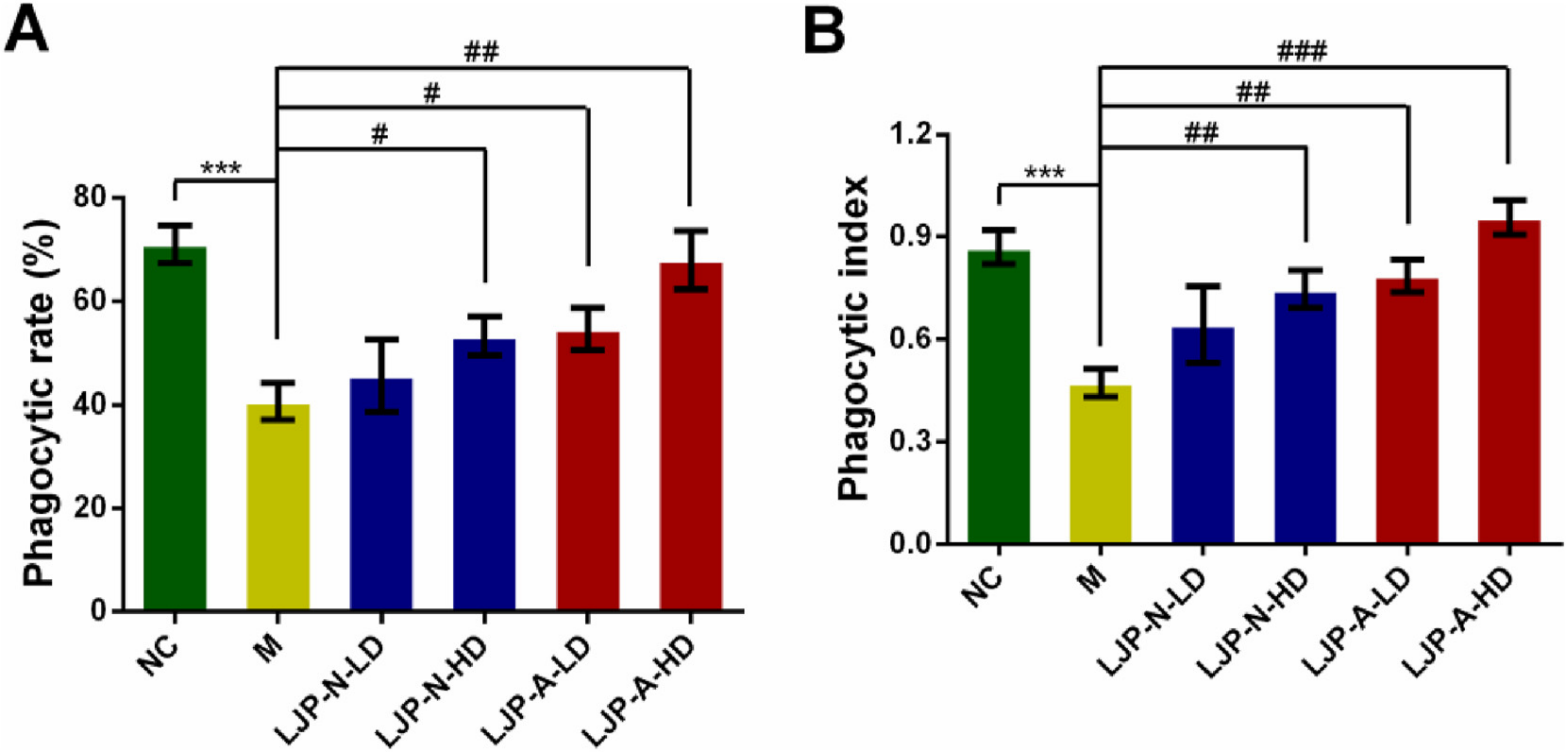
Effects of LJP fractions on macrophages phagocytosis indicated by India ink assay in CTX-treated mice. (A) Phagocytic rate (B) Phagocytic index. ∗∗∗, *P* < 0.001 *vs*. NC; #, *P* < 0.05, ##, *P* < 0.01 and ###, *P* < 0.001 *vs.* M. LD, 50 mg kg^−1^, HD, 200 mg kg^−1^.

Macrophage is the pivotal actor in the innate immune response, which plays a crucial role in human host defense system (Katsiari et al., 2010). The phagocytosis capability has been one of the key evaluation factors in studying the innate immune function. Phagocytosis was detected by injecting India ink through the tail vein. As shown in Figure 5, compared with that in the control group, the phagocytic rate (Figure 5A) and phagocytic index (Figure 5B) in CTX-treated model group were both significantly decreased (*P* < 0.001). After the treatment with LJP fractions, the phagocytic rate and phagocytic index were markedly increased by LJP-A (50 mg kg^−1^ and 200 mg kg^−1^), while the phagocytosis of macrophages in the LJP-N group was only significantly increased at the higher concentration (200 mg kg^−1^).

### 3.5 Effects of LJP fractions on NK cell cytotoxic activity

**Figure 6:**
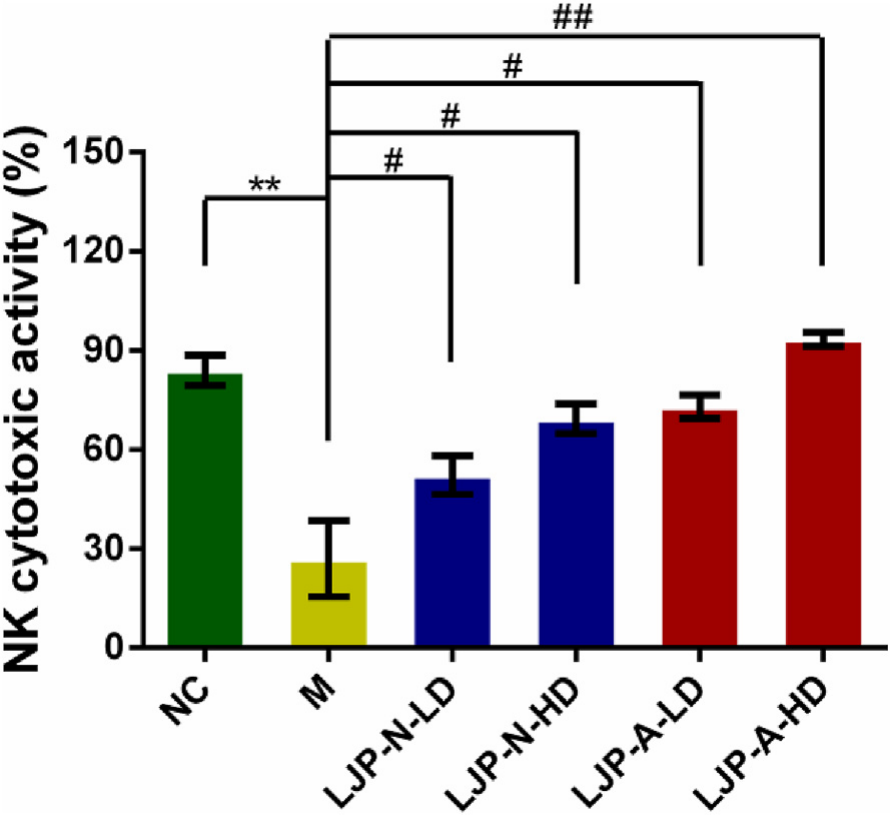
Effect of LJP fractions on the activation of mouse NK cells using MTT assay in CTX-treated mice. ∗∗, *P* < 0.01 *vs*. NC; #, *P* < 0.05 and ##, *P* < 0.01 *vs.* M. LD, 50 mg kg^−1^, HD, 200 mg kg^−1^.

NK cells possess a important immunomodulatory effects by recognizing the special cell ligands and killing harmful substances directly (Blanco et al., 2016). To analyze the cytotoxicity of NK cells, splenocyte cytotoxicity was determined against NK-sensitive tumor cells (YAC-1, the target cells of NK cells) in spleen cells isolated from the CTX-treated mice. As shown in Figure 6, compared with normal group mice, NK cells cytotoxic activity was significantly decreased in CTX model group mice (*P* < 0.01). After treatment with LJP fractions, LJP-N and LJP-A could both enhance the cytotoxic activity of NK cells with a dose dependent manner, which indicated that the LJP-N and LJP-A can modulate the innate immune response. Besides, LJP-A-fed mice showed a more significant increase in YAC-1 cell apoptosis than LJP-N-fed mice.

Under the evaluated concentrations, the immunomodulatory effects of LJP-A were significantly greater than those of LJP-N on the immune organ indices, cytokine (IL-2, IL-6), immunoglobulins, macrophage phagocytosis, and NK cell cytotoxicity. The greater activities of LJP-A are likely ascribed to its structural features, such as glycosidic linkage, monosaccharide composition, and molecular weight (Dong et al., 2018; Ferreira et al., 2015; Zeng et al., 2019).

Body weights of mice treated with LJP-A improved gradually, but there was no significance. While Wang et al. (2018) found that the body weights of mice treated with a polysaccharide from *Sarcodon imbricatus* (SIPS) improved body weights significantly compared with CTX group mice. Maybe the reasons were that after 3-day CTX injection continuously, CTX was additionally injected once a week and the time of mice treated with SIPS much more. Thymus and spleen indices are both important indicators of immune function, and Bai et al., 2020a, Bai et al., 2020b also found that a high molecular weight (377 kDa) polysaccharide obtained from longan pulp could significantly improve the spleen and thymus indices of CTX-induced immunosuppressed mice. Besides, Yu et al. (2014) and Zhang et al. (2012) reported that a polysaccharide (PSG-1) rich in GalA residues from *Ganoderma atrium*, with a high molecular weight of 1013 kDa, which could improve serum IgM and IgG levels in mice compared with CTX-induced immunosuppressed mice.

For glycosidic linkage, compared with α-Glucan (composed of α-glucose), β-Glucan composed of β-glucose glycosidic linkage, which can interact with dectin-1 receptor upon different types of immune cells to induce signal transduction that leads to trigger the MAPKs, T cells and NF-κB that produced cytokines and contributed to immune response (Ahmad et al., 2021; Brown et al., 2002; Kedir et al., 2022). For β-(1 → 4)-mannan polysaccharides, the types of glycosidic linkages might lead to large differences in the spatial structure of polysaccharide molecules, which would contribute to large differences in their immunomodulatory activities (Morris and Striegel, 2014; Tao et al., 2022). Pectic polysaccharides, rich in GalA residues, demonstrate considerable biological activities as the GalA residues can alter the polysaccharide properties and improve their water solubility (Tian et al., 2011). The immunomodulation activity of polysaccharides from *Ilex asprella* and *Litchi chinensis Sonn.* were positively correlated with their uronic acid content (Huang et al., 2016; Meng et al., 2018).

Besides, molecular weight is another important element that influences the immunomodulation effect of polysaccharides. For mannan molecules, the immunostimulatory activities of β-(1 → 3)-, α-(1 → 3)-, and α-(1 → 6)-mannans were reported with molecular weights in the range of 5–400 kDa, which suggested that the differences in molecular weight were not relation to other structural features (Ferreira et al., 2015; Lee et al., 2010; Tao et al., 2022). MOP-2 and MOP-3, isolated from the leaves of *Moringa oleifera*, had similar monosaccharide composition and glycosidic linkage types, but MOP-3 had better immunomodulatory activity, which might be ascribed to its higher molecular weight (Li et al., 2020). The average molecular weights of polysaccharide fractions ranging from 5 kDa to 400 kDa prepared from *Aloe* showed stronger macrophage-activating effect, compared with that of other fractions (Im et al., 2005). In this study, LJP-A containing more GalA residues (a type of uronic acids), exhibited stronger immunomodulatory effect than LJP-N, which is consistent with published studies. Moreover, polysaccharides can play as important prebiotics to promote the growth and metabolism of probiotics. Butyrate, one of microbial metabolites, can stimulate the activation of immune effector molecules to enhance the immunity of human intestinal mucosal. Thus, the prebiotic effects of LJP-A will be further studied in the future work.

## 4 Conclusions

In a conclusion, the results of the present study suggest that when compared with CTX group, LJP fractions, especially for LJP-A (200 mg kg^−1^), can enhance the immune function, improve atrophy of the lymphoid organs thymus and spleen (the increased approximately 1.8- and 1.6-fold), increase the phagocytic function of macrophages (increased approximately 1.7-fold), increase the secretion of cytokines (the levels of IL-2, IL-6 and TNF-α increased approximately 2.0-, 2.5- and 1.4-fold) and immunoglobulins levels (the levels of IgG and IgM increased approximately 1.7- and 1.2-fold) in serum, and enhance the cytotoxic activity of NK cells (increased approximately 3.5-fold). This study used a CTX-induced mouse model to demonstrate that the daily intake of a certain amount of LJP-A can be an effective way to inhibit CTX-induced decreases on immune function, which provides a basis for the research and development of LJP-A as a potent assistant immunomodulatory agent.

## Declarations

### Author contribution statement

Tao Zhang: Conceived and designed the experiments; Performed the experiments; Wrote the paper.

Hongping Liu: Conceived and designed the experiments; Analyzed and interpreted the data; Wrote the paper; Performed the experiments.

Pengcheng Ma; Jian Huang: Analyzed and interpreted the data.

Xinyu Bai; Ping Liu; Lei Zhu: Contributed reagents, materials, analysis tools or data.

Xun Min: Conceived and designed the experiments; Wrote the paper.

### Funding statement

This work was funded by the 10.13039/501100001809National Natural Science Foundation of China (No. 31760251, 32060035), the Program for High-Level Innovative “Thousand Level” Talents in Guizhou Province (Thousand Level), and the Program for Excellent Young Talents of Zunyi Medical University (No. 18zy-006).

### Data availability statement

Data will be made available on request.

### Declaration of interest's statement

The authors declare no conflict of interest.

### Additional information

No additional information is available for this paper.
